# Common architectures in cyanobacteria *Prochlorococcus* cells visualized by X-ray diffraction imaging using X-ray free electron laser

**DOI:** 10.1038/s41598-021-83401-y

**Published:** 2021-02-16

**Authors:** Amane Kobayashi, Yuki Takayama, Takeshi Hirakawa, Koji Okajima, Mao Oide, Tomotaka Oroguchi, Yayoi Inui, Masaki Yamamoto, Sachihiro Matsunaga, Masayoshi Nakasako

**Affiliations:** 1 Department of Physics, Faculty of Science and Technology, Keio University, 3-14-1 Hiyoshi, Kohoku-ku, Yokohama, Kanagawa 223-8522 Japan; 2 RIKEN SPring-8 Center, 1-1-1 Kouto, Sayo-cho, Sayo-gun, Hyogo 679-5148 Japan; 3 Graduate School of Material Science, University of Hyogo, 3-2-1 Kouto, Kamigori-cho, Ako-gun, Hyogo 678-1297 Japan; 4 Department of Applied Biological Science Faculty of Science and Technology, Tokyo University of Science, 2641 Yamazaki, Noda, Chiba 278-8510 Japan; 5 Present Address: Department of Integrated Biosciences, Graduate School of Frontier Sciences, University of Tokyo, 5-1-5 Kashiwanoha, Kashiwa, Chiba 277-8561 Japan

**Keywords:** Cellular imaging, Cell biology, Biological techniques, Imaging, Imaging and sensing

## Abstract

Visualization of intracellular structures and their spatial organization inside cells without any modification is essential to understand the mechanisms underlying the biological functions of cells. Here, we investigated the intracellular structure of cyanobacteria *Prochlorococcus* in the interphase by X-ray diffraction imaging using X-ray free-electron laser. A number of diffraction patterns from single cells smaller than 1 µm in size were collected with high signal-to-noise ratio with a resolution of up to 30 nm. From diffraction patterns, a set of electron density maps projected along the direction of the incident X-ray were retrieved with high reliability. The most characteristic structure found to be common among the cells was a C-shaped arrangement of 100-nm sized high-density spots, which surrounded a low-density area of 100 nm. Furthermore, a three-dimensional map reconstructed from the projection maps of individual cells was non-uniform, indicating the presence of common structures among cyanobacteria cells in the interphase. By referring to the fluorescent images for distributions of thylakoid membranes, nucleoids, and carboxysomes, we inferred and represented their spatial arrangements in the three-dimensional map. The arrangement allowed us to discuss the relevance of the intracellular organization to the biological functions of cyanobacteria.

## Introduction

Cyanobacterium *Prochlorococcus*, a unicellular organism with a size of less than 1 μm, is one of the most abundant photosynthetic organisms on the earth^1,2^. It contributes to nearly a half of the net primary production in marine ecosystem, such as oxygen production and carbon fixation^1,3,4^. In the cells, layers of thylakoid membranes with an approximate thickness of 100 nm associate with cell membranes and enclose cytosols containing nucleoids and carboxysomes. Recently, a new metabolism of *Prochlorococcus* cells, production of vesicles with size of diameters of 50–250 nm in cytoplasm and the release of them outside cells, was discovered and had a major impact on global biogeochemical cycles. Vesicles contain metabolic waste, which may be toxic compounds to be abandoned outside cells, or proteins and nucleic acids to be transferred to other cells^5,6^. A current model for mechanism of vesicle biogenesis proposes vesicle formation starts near the outer membrane with cargos of vesicles^6–9^. If cytoplasm of cyanobacteria cell is enclosed completely by several layers of thylakoid membranes, cargos of vesicles would be secreted to exterior of cells through complicated pathways including membrane fusion with thylakoid membranes.

Investigation of such biological activities requires structural data on the distribution of intracellular components of cyanobacteria. Transmission electron microscopy (TEM) applied to sliced sections of stained^10–13^ or frozen-hydrated cells^14,15^ has made significant contributions in this area to reveal the locations of thylakoid-membrane layers and carboxysomes. High-voltage TEM imaging has also visualized structure of cyanobacterial cells as a whole^14,16,17^. However, electron microscopy images of whole cells with diameters larger than 200 nm are under significant influences of multiple and inelastic scattering of electrons. In addition, radiation damage by a number of exposures of electron beams in tomography cause structural deformation^18^. Actually, most of the studies focused on thin periphery of cells such as thylakoid membranes and viruses on cell surface^14,17,19^.

From a small number of images for thin sections, it is difficult to imagine and discuss whole structures of cyanobacterial cells. Since biological cells are composed of well-organized and fragile cellular components, any other imaging technique complementary to TEM is necessary to visualize structures of whole cells without sectioning and chemical labeling of specimens. Since cell structures are somewhat different even in the same phase in the cell cycle, the technique is required to provide a large number of cell images enough to elucidate any common structures among cells in a specific cell-cycle phase and also to discuss heterogeneity.

**Figure 1:**
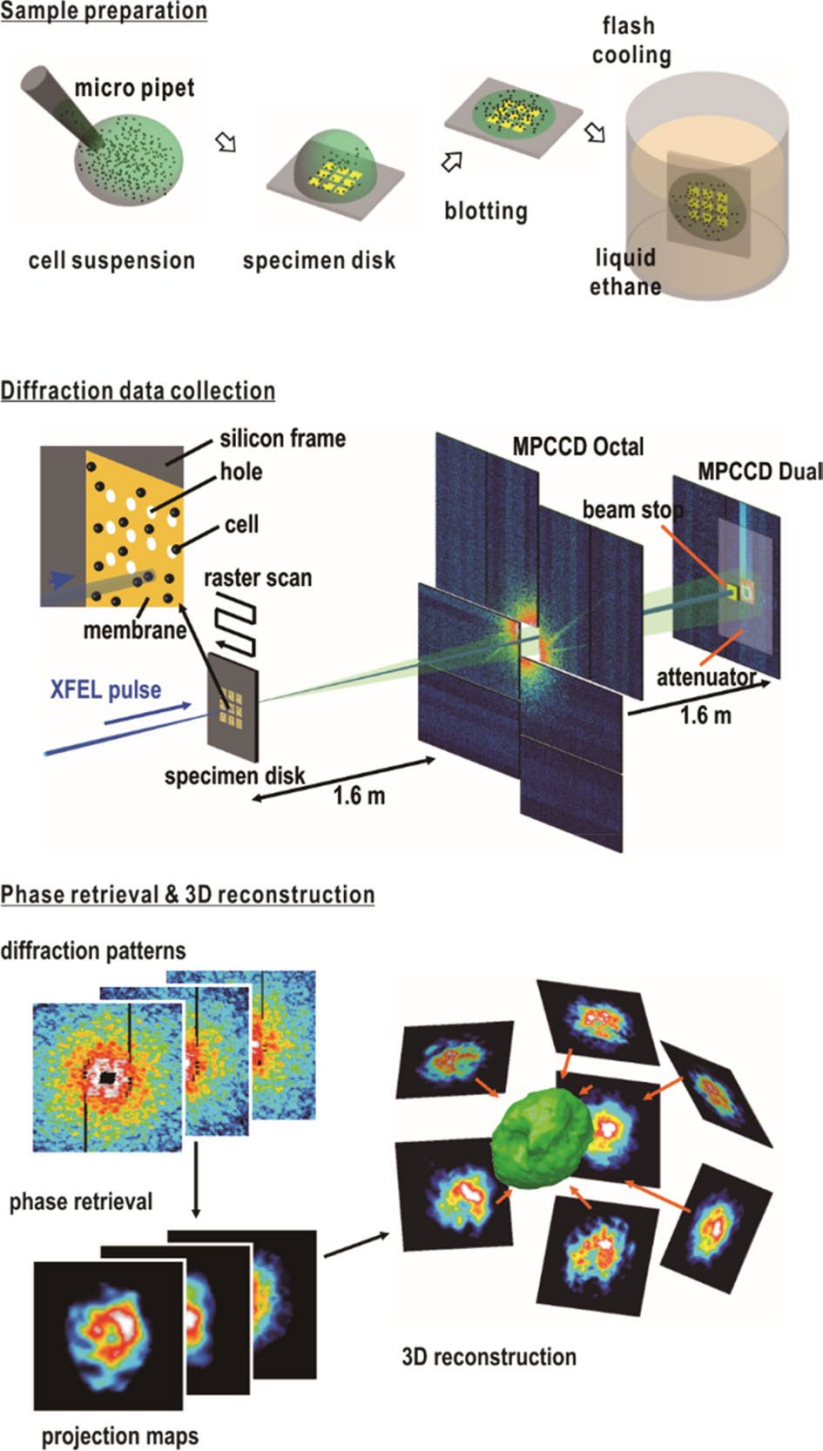
Schematic illustration of XFEL-XDI experiment and the subsequent structure analysis.

Compared with TEM, X-ray diffraction imaging (XDI) potentially allows us to visualize whole cells with sizes ranging from sub-micrometers to several micrometers at a resolution higher than that by light microscopy. Owing to the penetration power and little multiple scattering of short-wavelength X-rays in thick specimens^20,21^, XDI requires no chemical labeling and sectioning of specimens to possibly change any chemical and physical properties of cells. In XDI, a frozen-hydrated single cell is irradiated by a spatially coherent X-ray beam, and an electron density map projected along the direction of the incident X-ray is reconstructed only from a Fraunhofer diffraction pattern by applying the phase-retrieval (PR) algorithm^22^ (Fig. 1).

Recently, highly brilliant X-ray free-electron laser (XFEL) pulses, which have repetition rates of more than 30 Hz, allow collection of a large number of diffraction patterns of non-crystalline particles (XFEL-XDI) (Fig. 1)^23–25^. Single XFEL pulses provide diffraction patterns with a high signal-to-noise (SN) ratio up to a resolution of 30 nm from 1-μm sized cells and organelles before their destruction. Structures of cells visualized in XFEL-XDI is almost free from radiation damage at the atomic level, as demonstrated in protein crystallography using XFEL^26^. Previous structural studies have demonstrated that a number of projection maps are useful to investigate varieties of internal structures in non-crystalline particles in both biology and material sciences^27–33^. Therefore, XFEL-XDI has potential to structurally characterize cells in a cell-cycle phase.

In addition to the ordinary analysis in XFEL-XDI, a challenging analysis is the construction of an averaged three-dimensional (3D) structure of cells from a number of projection maps of cells with very similar sizes in different orientation against the direction of X-ray beam, as in single particle analysis in TEM. If cells possess any common structural features, they will appear in the averaged 3D map. Otherwise, the 3D map will be filled by uniform density. In addition, sectional view of the 3D map will be advantageous to visualize low-electron-density features with high contrast, which are often buried in other high-density features and invisible in projection maps. This challenging approach would be suitable to find any common structural features including low density regions inside cells.

Here, we present structures of *Prochlorococcos marinus* MED4 cells in the interphase by XFEL-XDI. Projection electron density maps of individual cells suggested structural characteristics common among cells, and therefore encouraged us to reconstruct a 3D map averaged among cells from the projection maps. The 3D map represented a common arrangement of cellular components in *Prochlorococcus* cells, which were identified with a help of fluorescent microscopy imaging. Based on the projection and 3D maps, we discussed the biological roles of the identified components of *Prochlorococcos*, including the production and release of vesicles.

## Results

First, we show fluorescence microscopy images to address rough locations of major components in *Prochlorococcus* cells. Next, we describe intracellular geometrical characteristics in projection electron density maps obtained by XFEL-XDI experiments (Fig. 1). In addition, we present an 3D map reconstructed from the projection maps to elucidate common structures among cells.

### Fluorescence microscopy imaging of *Prochlorococcus* cells

**Figure 2:**
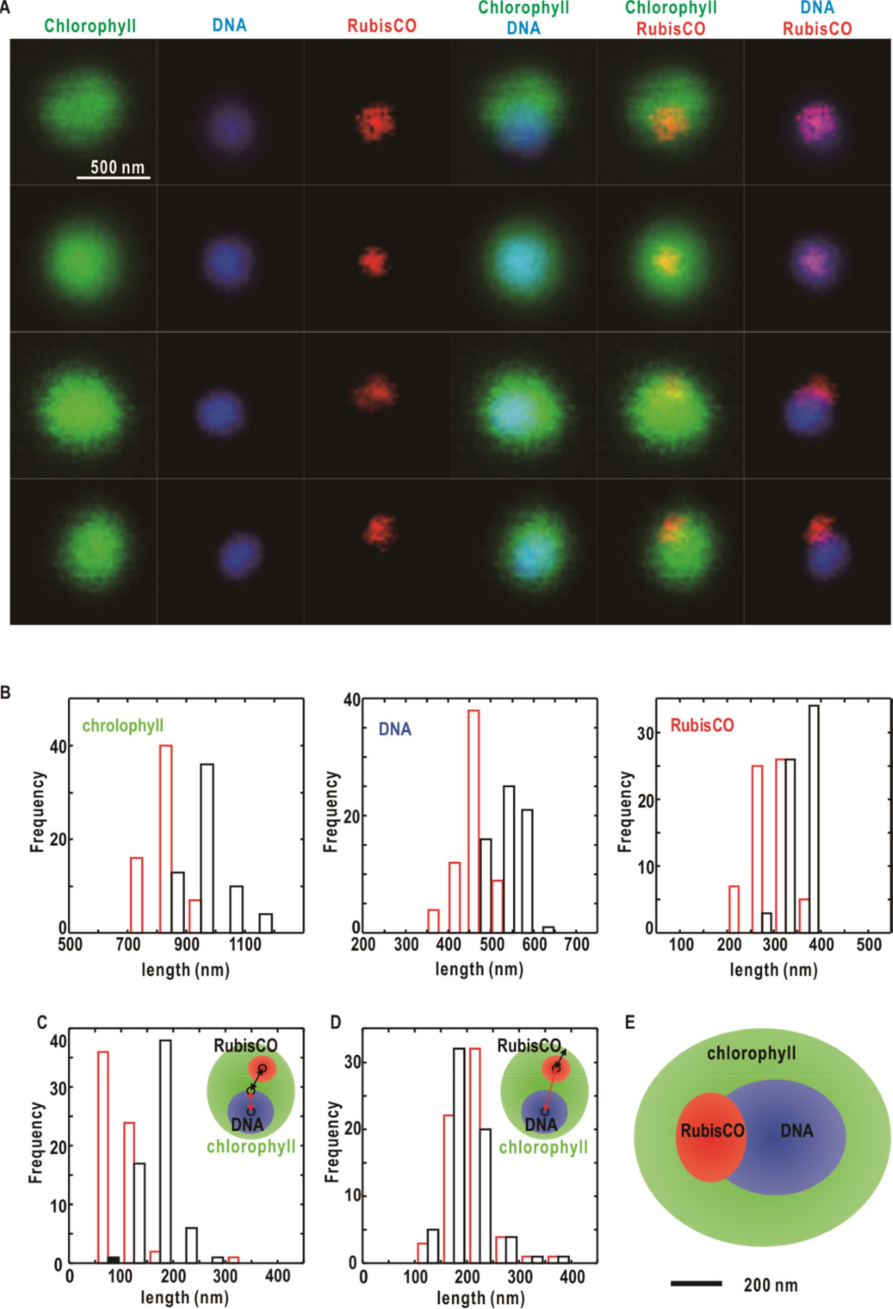
Fluorescence microscopy investigation of cellular components*.* (**A**) Representative fluorescence microscopy images of *Prochlorococus* cells showing the distributions of chlorophylls (green), DNA stained with DAPI (blue), and immuno-stained RubisCO (red). The three columns in the right are merged images of the three. (**B**) Frequency distribution of component sizes in fluorescence images of the three components. The red and black bars show the length frequencies for the long and short axes, respectively, when each image was roughly approximated as an ellipse circle. (**C**) Frequency distribution of distances between the centers of chlorophyll and DNA images (red bars) and those between chlorophyll and RubisCO images (black), as illustrated in the inset. (**D**) Frequency distribution of the distances between the centers of DNA and RubisCO images (red) and those between the center of RubisCO image and envelop of chlorophyll image (black). (**E**) Schematic illustration of the average distribution of chlorophyll, DNA, and RubisCO in *Prochlorococus* cells based on panels (**B**–**D**).

Distribution of three components, chlorophylls, deoxyribonucleic acid (DNA), and ribulose-1,5-bisphosphate carboxylase/oxygenase (RubisCO) molecules, in *Prochlorococcus* cells in the interphase were visualized by fluorescence light microscopy (Fig. 2A). Auto-florescence images of chlorophyll molecules in thylakoid membranes indicated a uniform chlorophyll distribution in spheres or hemi-spheres with diameters of 700–900 nm (Fig. 2B). According to several TEM images of sliced sections of cyanobacteria cells, in which layers of thylakoid membranes are distributed along the envelope of cells^18^, the auto-fluorescence images of chlorophyll molecules give approximate sizes and shapes of *Prochlorococcus* cells.

DNA stained with 4′,6-diamidino-2-phenylindole (DAPI) was distributed uniformly in spheres with diameters of 400–500 nm (Fig. 2B), the centers of which were mostly located within 200 nm from those of chlorophyll spheres (Fig. 2C). The distribution of ribulose-1,5-bisphosphate carboxylase/oxygenase (RubisCO), a major component in carboxysomes^34^, was visualized by immune-staining. Their fluorescence was limited within irregular-shapes with dimensions of 200–400 nm (Fig. 2B). The center-to-center distances between DAPI and RubisCO distributions indicated that carboxysomes were located on the periphery of DAPI spheres (Fig. 2C,D). The centers of RubisCO regions were located approximately 200 nm away from the outer edges of chlorophyll regions (Fig. 2D).

The fluorescence images allowed us to roughly illustrate the organization of the three major components (Fig. 2E). Nucleoids and carboxysomes were enclosed in thylakoid membranes. They were distributed with little overlaps, and their centers were located approximately 200 nm away from the center of thylakoid membranes.

### Diffraction patterns and projection electron density maps

In four different XFEL-XDI experiments, a number of diffraction patterns were collected by scanning specimen disks, on which frozen-hydrated *Prochlorococcus* cells were dispersed, against XFEL pulses (Fig. 1). Cells were mostly in the interphase of the cell cycle as measured by flow cytometry (SI Appendix, Fig. S1). Diffraction patterns of cells in the mitotic phase could be excluded because their strong diffraction intensities due to the almost duplicated cell contents caused an overflow of detector pixels in small-angle diffraction region. In addition, larger cell sizes in mitotic phase made interference patterns finer.

**Figure 3:**
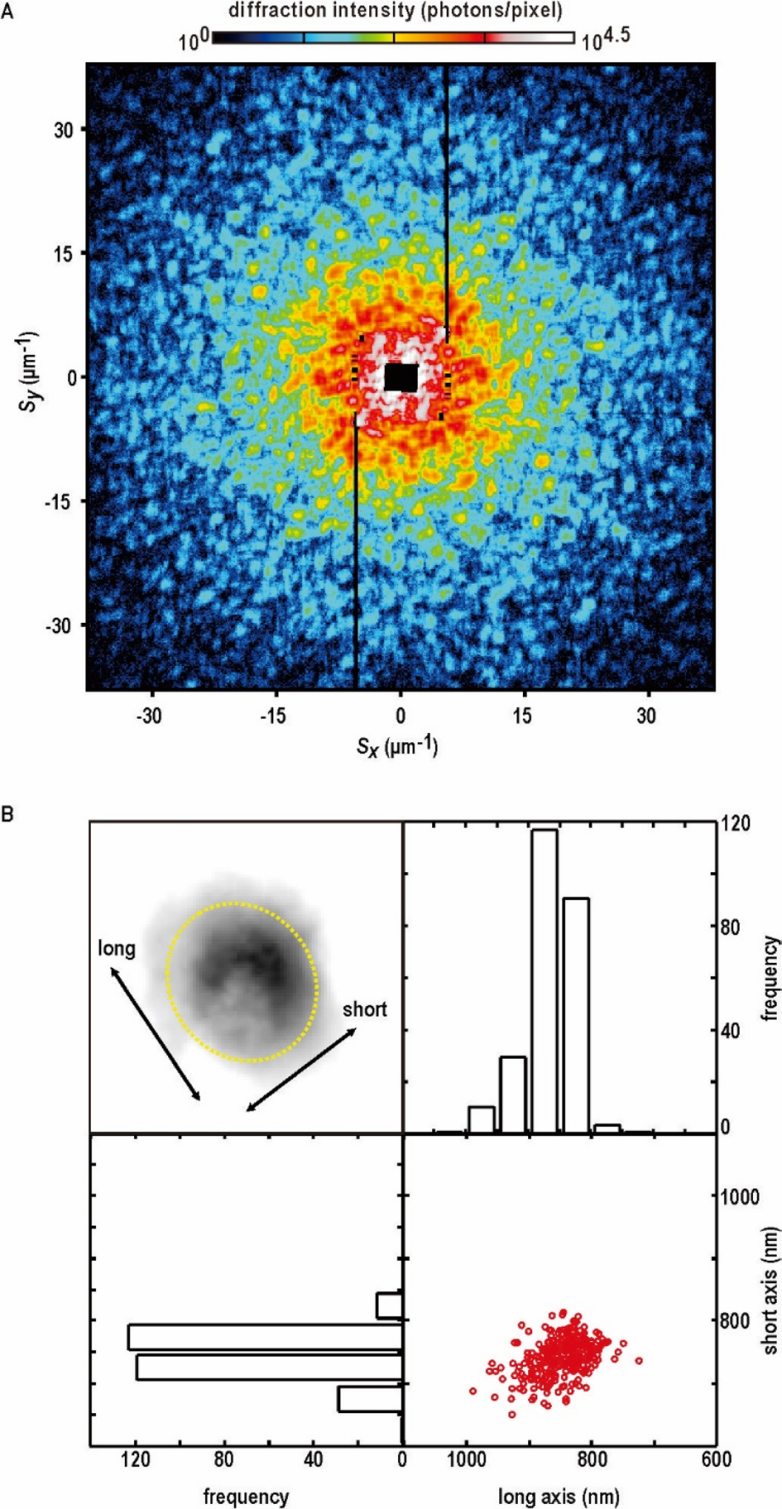
Representative results of XFEL-XDI experiments. (**A**) Representative diffraction pattern from a single *Prochlorococus* cell. *S* is the scattering vector defined as $S = {{2\sin \theta } \mathord{\left/ {\vphantom {{2\sin \theta } \lambda }} \right. \kern-\nulldelimiterspace} \lambda }$, where 2*θ* is the diffraction angle and *λ* is the X-ray wavelength. (**B**) The frequency distribution of lengths of long (upper right) and short axes (lower left) of projection electron density maps (upper left) and their correlation (lower right).

From tens of thousands diffraction patterns of cyanobacteria, we selected carefully high-quality diffraction patterns by evaluating maximum spatial frequency, SN ratio and sizes of interference peaks. As a result, we selected 1061 patterns, which were composed of interference patterns with high SN ratio up to a spatial frequency of 40 μm^−1^ (corresponding to a resolution of 25 nm in real space) (Fig. 3A and SI Appendix, Fig. S2A). Interference peaks were approximate diameter of 1.3 μm^−1^, and the size was comparable with those expected as the reciprocal size of the *Prochlorococcus* cell (approximately 800 nm) measured by an optical microscope^35^. After PR calculations of 1061 patterns for the subsequent analysis. we selected 293 projection electron density maps, which satisfied a criterion regarding the convergence in PR calculation (SI Appendix, Fig. S2B,C). The selected maps displayed crystallographic *R*_*F*_ factors of smaller than 30% (SI Appendix, Fig. S2D), and the effective resolution were higher than 80 nm (SI Appendix, Fig. S2E). In the initial stage of each PR calculation, the size and shape of PR map was roughly estimated from the autocorrelation functions directly calculated from the diffraction pattern. The selected maps were located in the most frequent region in solution space of a large number of PR calculations (SI Appendix, Fig. S3).

Projection maps display electron density inside cells excess from the average electron density of vitreous ice because of the contrast modulation, i.e. only the electron density difference between the cells and vitreous ice contributes to X-ray diffraction^35^ (SI Appendix, S2). Therefore, both vitreous ice outside cells and cell regions with electron densities comparable with the average density of vitreous ice display almost zero in the projection maps, and then, high electron density region inside cells appear with a high contrast rather than cell shapes. Diffraction patterns from cells in vacuum is dominated by the scattering from the shape, and the boundary of cell is clearly defined^32^. However, we chose the cryogenic experiment to visualize cells without boil of water, adiabatic expansion and drying, although the cell border is blurred by the electron density contrast^21^.

**Figure 4:**
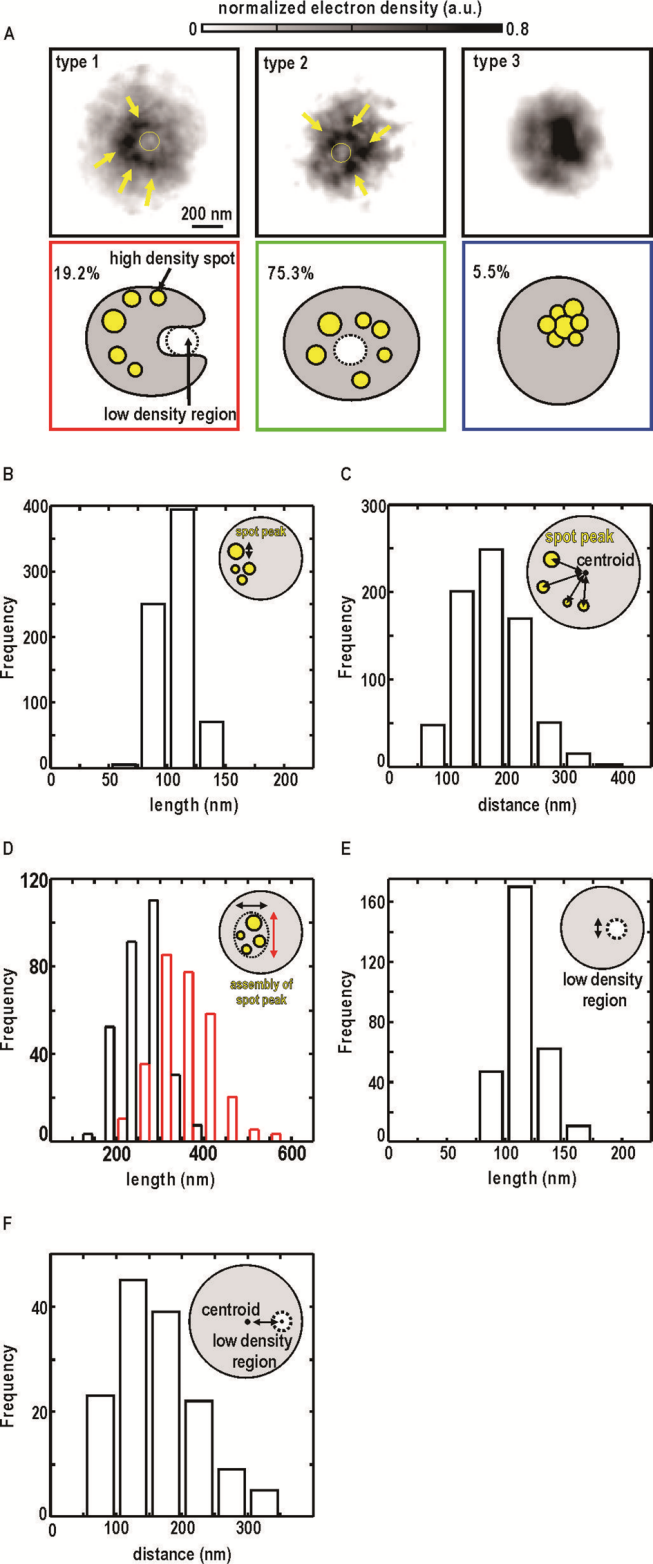
Classification of projection maps with respect to characteristics internal structures. (**A**) Representative electron density maps classified into three types. Arrows and dotted circles in the upper panels indicate spot peaks with electron density values 80% higher than the maximum in each map, and low electron density regions, respectively. Lower panels schematically illustrate density distribution in each type. Percentages of each map type are indicated. (**B**) Size distribution of high-density spots, and (**C**) distance between the centroids of high-density spots and whole density distribution. (D) Frequency distributions of the sizes of density regions higher than 15% of the maximum in each map of type 1 or 2. (**E**) Size distribution of low-density region, and (**F**) distance between the centroids of low-density region and whole density distribution.

The rough boundaries of the projection maps could be approximated as ellipses with average dimensions of 900 × 800 nm (Fig. 3B) as reported in the previous studies^35^. The wavy-envelope of the projection maps reconstructed by CDI was quite similar to that observed in fluorescence images of chlorophyll molecules shown in Fig. 2B. Projection electron density maps implied a non-uniform distribution of cellular components. We classified the projection maps roughly into three (types 1, 2, and 3) with respect to the locations of characteristic density regions described below (Fig. 4A and SI Appendix, Fig. S5). Maps of types 1 and 2, accounting for 94.5% of all, were characterized by a set of several spots with densities higher than 80% of the maximum (arrows in Fig. 4A), and also by a low-density region (doted circles in Fig. 4A) surrounded by a C-shaped density in type-1 or semicircular density in type-2 maps. In type-3 maps, several high-density spots seemed to be assembled together, and low-density regions were almost absent.

In type-1 and type-2 maps, high density spots had approximate sizes of 100 nm (Fig. 4B). The spots were located 100–300 nm away from the centers of projection densities (Fig. 4C), and were arranged along C-shaped or semicircular densities of more than 60% of the maximum (Fig. 4A). They formed assemblies with sizes of 300–400 nm (Fig. 4D). Low density regions found in type-1 and type-2 maps had an average diameter of 100 nm (Fig. 4E), and were located 100–300 nm away from the centers of projection maps (Fig. 4F).

### Averaged 3D structure

**Figure 5:**
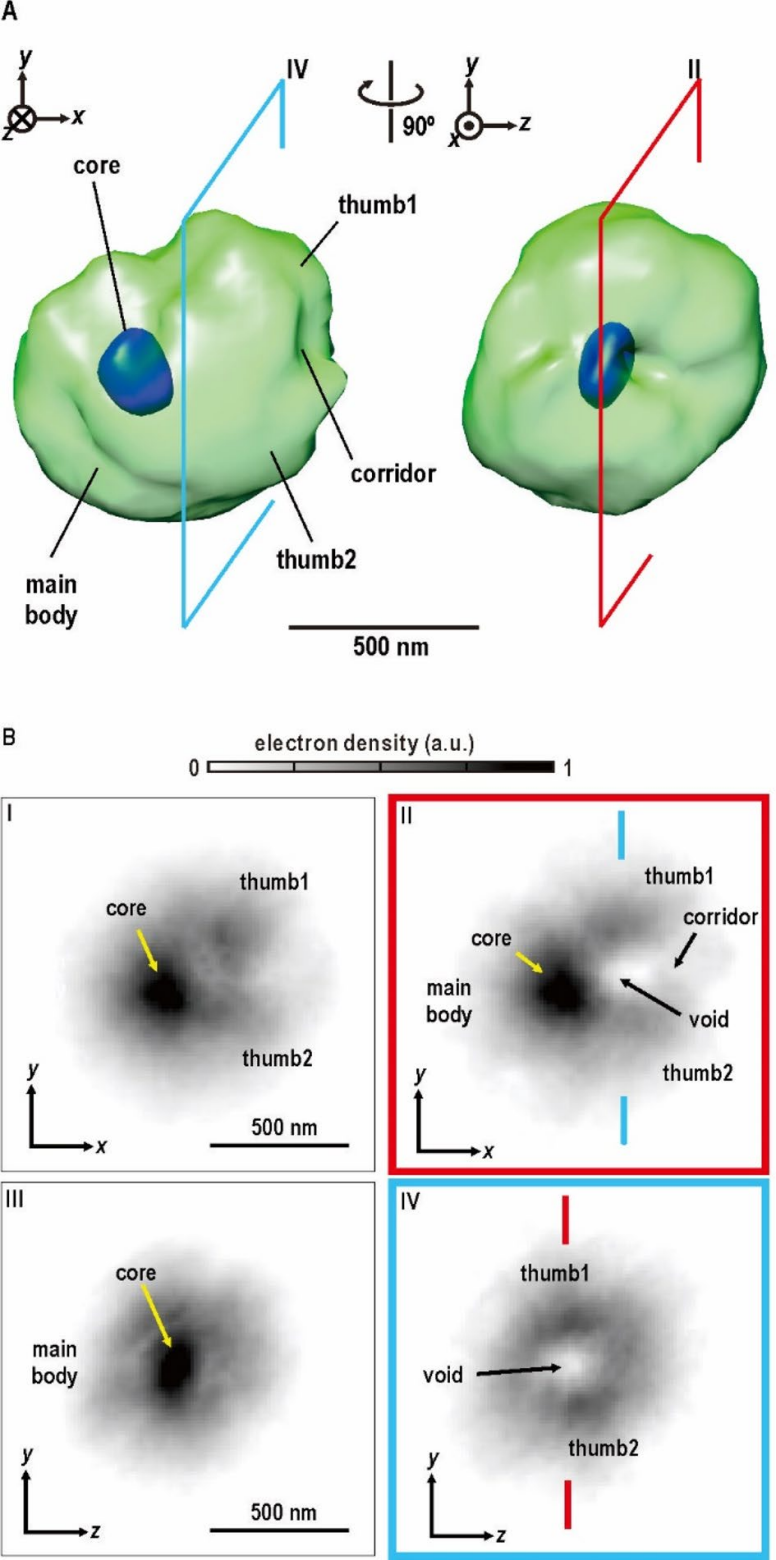
Averaged 3D structure among individual cells. (**A**) 3D electron density map reconstructed from 293 projection maps. The green and blue surfaces are contoured at 20% and 80% of the maximum value in the 3D map. The 3D map was illustrated using the UCSF Chimera suite ^70^. (**B**) Projection (I, III) and cross-sectional (II and IV) views of the 3D electron density map (cross-sections II and IV are indicated in panel **A**). Characteristic densities were labeled as described in the main text.

When the projection maps are viewed at effective resolution of better than 80 nm, electron density distribution indicated heterogeneity among the 293 cells in the interphase even when we carefully selected diffraction patterns and projection maps described above. On the other hand, at a low resolution, a rough classification regarding the arrangements and sizes of major structural components was possible, implying the presence of common structures and intracellular organization patterns among the cells (Fig. 5). The application of single particle analysis to 293 projection maps would smooth the structural heterogeneity observed in the high-resolution maps and emphasize common structural characteristics viewed at a low resolution. Therefore, a 3D density map reconstructed by such an analysis would be useful to elucidate the common characteristics.

A 3D map (Fig. 5A) was reconstructed as an ellipsoid sphere without bias from heavily preferred orientation of cells against the direction of the incident X-rays. But, the slightly uneven distribution on the orientational distribution suggested, the long axes of cyanobacteria cells tended to be parallel along the membrane of specimen disk (SI Appendix, Fig. S6A). As the electron density profiles in the sectional views display similar gradients between the low and high regions both in the directions parallel and normal to the preferential orientation (Fig. S6B), the reconstructed map is probably from blurring by the slightly uneven orientation.

The effective resolution of the 3D map was estimated to be 152 nm from a Fourier shell correlation (FSC) curve for two 3D maps reconstructed from independent half datasets (SI Appendix, Fig. S6C). Since the FSC from the two independent data set reflects the heterogeneity of cells, cells have similar structures viewing at the resolution. In addition, as the orientation of each PR projection map was determined in the 3D reconstruction process, we compared the PR map and the projection map calculated from the reconstructed 3D map at the determined orientation. Then, the correlation coefficient between the maps was greater than 0.95 at a pixel resolution of 152 nm over the PR maps used in the reconstruction (SI Appendix, S3). This result implies that each PR map used in the reconstruction has significant contribution to the reconstructed 3D map, and also that structural features in a few projection maps have little bias on the 3D map.

As FSC curve relies on the variety and quality of projection maps, here we assessed maps in terms of phase errors. The averaged phase-retrieval transfer function (PRTF) among 293 PR maps, a measure of the degree of phase error, displayed high reliability (larger than 0.9) of phase set up to a resolution of 136 nm (SI Appendix, Fig. S2F). In addition, the structure amplitudes, which were calculated from each projection map of the 3D map, were consistent with the observed amplitudes beyond the effective resolution from FSC (SI Appendix, Fig. S7C). These evidences imply that cyanobacteria at the interphase have almost identical structures, and the reconstructed 3D map present an averaged structure viewed at a resolution of 152 nm.

When 3D map was visualized in the range greater than 20% of the maximum, an anisotropic distribution of electron densities became apparent (Fig. 5B). The density was distributed in an ellipsoid sphere with approximate dimensions of 900 × 800 × 800 nm as expected from the projection maps. The half of the map was occupied by a main body of a hemi-sphere shape accompanied with two bulges, designated ‘thumb1’ and ‘thumb2’. Between the thumbs, we found a low-density region named ‘void’ (typically seen in projection I and section II in Fig. 5B) and a low-density path ‘corridor’ connecting the to the cell surface.

The main body with an approximate dimension of 550 nm could be interpreted to come from C-shaped and/or semi-circular distribution of densities observed in projection maps of types 1 and 2. Their centers were located 200 nm away from those of the cells. The main body contained a high-density region (exceeding 80% of the maximum density) designated ‘core’ of an ellipsoid sphere with approximate dimensions of 250 × 150 × 150 nm. The distance between the centers of the main body and core was approximately 150 nm, and was consistent with the distribution maximum of high-density spots (Fig. 4C).

The thumbs 1 and 2 protruding from the edge of the main body by 200–300 nm (projection I and section II in Fig. 5B) predominantly originated from the edges of C-shaped regions in projection maps, and displayed densities comparable to those observed in most part of the main body except the core. As seen in section-IV in Fig. 5B, both thumbs formed a short and curved wall to surround the void and corridor. The void with a dimeter of 150 nm was located approximately 100 nm away from the center of the cell. The electron density in the void was as low as that of vitreous ice surrounding cells according to the contrast variation theory^35^ (SI Appendix, S2). Both the void and corridor originated from low density regions observed in types1 and 2 projection maps. In type-3 maps (Fig. 2C and SI Appendix, Fig. S5), low density region was unclear, probably because it overlapped with high density regions when viewed from the direction of the x-axis, as in projection III.

## Discussion

The present XDI study revealed structural characteristics shared by *Prochlorococcus* cells in the interphase from projection maps, as well as common structural components among the 293 cells from a reconstructed 3D map. By inspecting projection and 3D maps together with the fluorescence microscopy images, we discuss cellular structures in the interphase and their relevance to biological activities of cyanobacteria.

### Organization of cellular components

By referring the fluorescence microscopy images (Fig. 2) as well as the electron microscopy images reported previously^10–15^, we identified cellular components yielding high and low electron-density regions (Figs. 3, 4, 5). Since the shapes and size distribution of whole cells in projection maps (Fig. 4) are similar to those observed in fluorescence images of chlorophyll molecules (Fig. 2), the regions just inside the outer edges of the projection maps can be assigned as layers of thylakoid membranes. Areas lacking thylakoid membranes in the C-shaped electron density distribution (Figs. 4A and 5A) could contribute to hemi-spherical distributions of chlorophyll molecules in fluorescence images (Fig. 2A). The incomplete enclosure of thylakoid membranes in the 3D map is consistent with reported TEM images^10,12–15^. Although thylakoid membranes observed in several TEM images of sectioned cyanobacteria cells^10,11,15^ completely enclosed cytosol, those could be interpreted as images, for instance, of slices along the direction of section-IV in Fig. 5.

The locations, sizes, and distributions of high-density spots (Fig. 4) were similar to those observed in fluorescence images of immuno-stained RubisCO (Fig. 2) and carboxysomes observed in TEM images^15,34^. Since carboxysomes are densely filled with large amounts of RubisCO, carbonic anhydrase, and shell protein molecules^34,36^, their electron densities would be high enough to yield high density spots. Therefore, we assigned high density spots as carboxysomes. The frequency distribution regarding the distances between the spots and cell centers (Fig. 4C) suggests that carboxysomes are located near the thylakoid membranes rather than near central regions of cells. The same tendency was seen in TEM^13,15^. Then, the core in the 3D map would represent an area, where carboxysomes are most frequently and densely distributed. Carboxysomes are distributed along layers of thylakoid membranes and contribute to the increase in apparent electron densities. The location of carboxysomes near thylakoid membranes is geometrically advantageous for the production of ribulose 1,5-bisphosphate through the phosphorylation of ribulose-5-phasphate immediately after their synthesis in thylakoid membranes^37^.

DNAs were distributed as a nucleoid within a sphere with a diameter of 500 nm as demonstrated by fluorescence microscopy for DAPI-stained cells (Fig. 2). In TEM images of frozen-hydrated *Prochlorococcus marinus* MED4 cells^10–15^, DNAs appear unclear with a low contrast, probably due to their sparse distribution and also the scattering cross section for electrons comparable with those of cytosol components^38,39^. When the 1.66-Mbp genome of *Prochlorococcus* cell with a mass of 1.7 × 10^–15^ g^40^ is distributed uniformly within a 500-nm sphere in cytosol (Fig. 2B), the electron density at a mass density of 0.026 g cm^−3^ is 7.5 electrons nm^−3^, which is much smaller than that of water (334 electrons nm^−3^)^41^, as calculated by referring to the electron density of nucleic acid molecules (550 electrons nm^−3^ at a mass density of 1.9 g cm^−3^)^41^. Since genomes are physically associated with proteins, the density of nucleoid region would be as low as water. Therefore, considering the low electron density contrast and its appearance in the fluorescence images, nucleoid distribution would overlap the low-density void.

### Relevance of cellular organization to biological activity of *Prochlorococcus*

**Figure 6:**
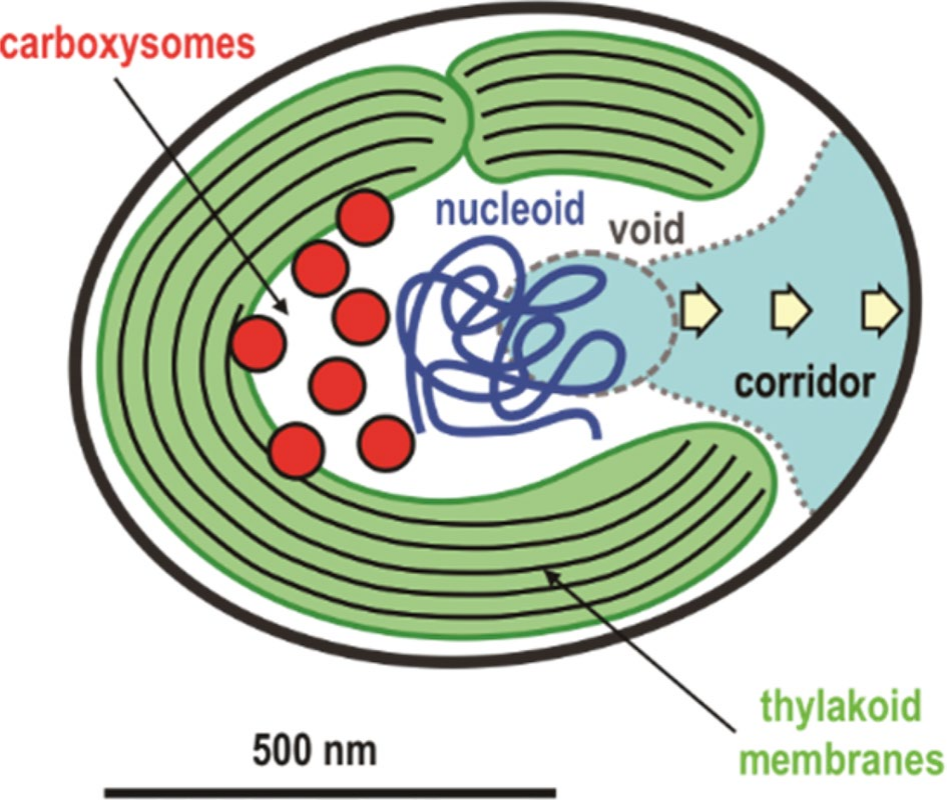
Model showing an arrangement of fundamental cellular components in the *Prochlorococcus* cells.

Based on the assignment described above, the organization of the three major components in *Prochlorococcus* cells is schematically illustrated in Fig. 6. Layers of thylakoid membranes are distributed along the cell membrane with a gap at one side. In this regard, we observed C-shaped structures both in the 3D map of *Cyanidioschyzon merolae* cell and in the projection map of a single chloroplast^30,42^. According to the endosymbiotic theory, cyanobacteria are thought to be the origins of chloroplasts in eukaryotic bacteria. Therefore, the similar C-shaped density implies that hemi-spherical structures in cyanobacteria are conserved in chloroplasts of *Cyanidioschyzon merolae*. Since cyanobacteria and red algae rotate freely in the living medium, the hemi-spherical layers of thylakoid membranes would be more advantageous than the grana found in higher plants with a fixed orientation to efficiently accept sun light for photosynthesis.

*Prochlorococcus* cells release 50–250 nm-sized vesicles to exclude metabolic waste or to transfer surpluses of proteins and nucleic acid molecules to other cells^5^. In physiochemical models, the presence of reservoir regions for the assembly of cargo molecules has been proposed^43–46^. After a saturation of molecules in assembling biomolecules, surplus molecules continue to be diffused in cytoplasmic reservoir. In this regard, a gap of a thylakoid membrane is found in a tomographic TEM imaging for slices of cyanobacteria^15^. The gap may correspond to an exit of the corridor found in the present 3D map. However, the void region was missed possibly due to the low contrast and SN ratio at the centers of cells in the TEM images, where multiple scattering of electrons becomes significant.

As the relatively low electron densities in the void and corridor can be interpreted as low concentrations and/or average electron densities of substances, the difference in electron densities between the void-corridor region and other cytoplasmic regions might result in chemical-potential gradient between them. Therefore, we speculated that the void-corridor region might be a cytoplasmic reservoir for proteins, nucleic acids, and metabolic waste, and also as a region of cargo transportation from the cytoplasm to cell surface without obstruction by any organelles. The void-corridor region might constitute a place to gather molecules near cell surface without any additional energy expenditure. This speculation may be a clue to understand the current model for the mechanism of bacterial vesicle biogenesis, which proposes that formation of vesicles starts near the outer membrane with cargos^6–9^.

Of course, the possibility is necessary to be examined by other imaging techniques. In our XDI experiment, diffraction intensity is sensitive to electron density distribution and contrast in cells excess from the average electron density of vitreous ice in projection maps (SI appendix, S3). Thus, the projection maps in types 1 and 2 would lead us to find the void and corridor regions. Tomography XDI experiment at cryogenic temperature may be more suitable to visualize the 3D structures of single cells at higher resolution than the averaged image^42^. High-voltage TEM could be one of solutions for visualize the structure of thick specimens. However, because scattering cross sections of light atoms composing cells are comparable for high-energy electron beam, it is difficult to distinguish the void and corridor regions with low concentrations of substances. In addition, in fluorescent microscopy images, these regions would be buried in other features due to sum of fluorescence intensities along depth direction and be blurred by the point spread of fluorescence.

Radiation damage by synchrotron X-ray is serious for sample measured at room temperature^47^, in contrast to the cryogenic condition^42^. For instance, the maximum tolerated dose for hydrated cells is 10^5^ Gy in X-ray fluorescent microscopy, because of the destructive diffusion of free radicals produced from water molecules^48^. In small-angle X-ray scattering, protein molecules solution shows excessive aggregation for doses above ~ 400 Gy^49^. In contrast, when using XFEL pulses with the 10-fs duration and the intensity of 10^10–11^ photons/pulse provided at SACLA, specimens are almost free from radiation damage as demonstrated by a crystallographic study^26^. Therefore, the variation of electron density, such as void and corridor, in the averaged 3D structure is not caused by XFEL irradiation but is intrinsic and common structure of cyanobacteria cells. XFEL-XDI is suitable to visualize structures of radiation-sensitive specimens.

### Reconstruction of 3D image of cells with heterogeneity

Despite the heterogeneity in fine structures among cells, which is typically seen in the distribution of high-density spots, the projection maps can be roughly classified into three types by inspecting the global distribution of dense and sparse electron density regions. Furthermore, non-uniform density distribution in the reconstructed 3D map suggest the presence of characteristic structures common among cells as described above.

According to the single particle analysis for projection maps of particles with the same structures, the achievable 3D resolution *d* is roughly estimated as

1 $$ d = \frac{\pi D}{{N,}} $$

where *N* and *D* are the number of orientations and the size of the sample, respectively^50^. Therefore, for particles of similar structure with *D* = 900 nm, projection maps (*N* = 293) sampled at different orientations yield an achievable resolution of 9.6 nm in the 3D map. However, the actual resolution achieved is limited to 152 nm. Because the comparison between the observed structure amplitudes and those calculated from the 3D map (SI appendix, Fig. S7C) indicated that the 3D map included less phase retrieval errors beyond the effective 3D resolution, the decrease of the 3D resolution is mainly caused by the structural heterogeneity of specimen particles at higher resolution^51,52^. Actually, correlation coefficients between projection maps and those projected from the 3D map are lower at higher pixel resolution than 152 nm (SI appendix, Fig. S7B). On the other hand, viewed at a resolution of 152 nm, non-uniform distributions of electron densities in individual cells are comparable to each other. This similarity yields the 3D map, in which possible territories of cellular components with sizes larger than the effective resolution and/or large contrast in electron densities can be visualized.

Although the number of individual cells in each orientation appears to be small, in the 3D reconstruction procedure, each Fourier slice calculated from the projection maps is assembled in the 3D Fourier space and the ensemble average of the projection maps is performed as voxel-by-voxel in the Fourier space. We calculated the number of projection maps assigned to each Fourier voxel, which was prepared at the Nyquist interval (SI appendix. Fig. S8). That indicates that at least ~ 30 projection maps were averaged up to the resolution estimated by FSC. Although the number of averaged cells estimated through above consideration is not large in the statistical view, the reconstructed map is expected to provide tendency of the common structures and approximated size scale of heterogeneity.

The present study demonstrated that the 3D reconstruction from a number of projection maps of cells in almost synchronized cultivation can emphasize large and structures common among cells. Therefore, while TEM images of sliced specimens visualize the details of sliced cell structures, XFEL-XDI can provide a global perspective on internal structures of cells without modification. By improving the efficiency in data acquisition, a larger number of high-quality diffraction patterns will provide an ensemble of structures, and allow reliable classification of PR maps by, for instance, manifold learning as demonstrated in our XFEL-XDI study on metal particles^25^. In addition, it is necessary for future studies to develop more sophisticated method to address the reliability of PR maps and reconstructed 3D maps by introducing probability distribution, Bayesian inference and refinement of phase set. The complementary use of XFEL-XDI with fluorescence microscopy, which provides the distribution of chemically labeled biomolecules, reveals intracellular distribution of cellular components, and thereby provides insights into their biological functions.

## Materials and methods

### Cultivation of cyanobacteria

*Prochlorococcus marinus* MED4 strain NIES-2087 was purchased from the National Institute for Environmental Studies, Japan. Cells were cultured in the PRO-99 medium^53^ until they reached the stationary phase. The amount of DNA contained in cells was examined by flow cytometry analysis using a Cytoflex (BeckMan Cortar, USA) after staining with SYTOX green (Invitrogen, Carlsbad, CA, USA) (SI Appendix, Fig. S2).

### Fluorescent microscopy imaging

A 500-µL cell suspension from the culture was centrifuged for 5 min at 15,300×*g*. The pellet was mixed with 100 µL of fixation buffer, containing 10% (v/v) dimethyl sulfoxide (DMSO), 1% paraformaldehyde, and 0.4 mM NaOH/methanol, and the mixture was incubated at 253 K for 5 min. After the incubation, 200 µL of iced methanol (99.5%) was added to the mixture, followed by centrifugation for 5 min at 15,300×*g*. The pellet formed was mixed with 50 µL of iced methanol (99.5%). The mixture was centrifuged for 5 min at 15,300×*g*, and the pellet was mixed with phosphate buffered saline (PBS). The resultant cell samples were used in immunofluorescence imaging.

Some portion of the sample prepared as described above was suspended in PBS, and the suspension was centrifuged for 3 min at 800×*g*. The pellet was collected and resuspended in 30 μL of PBS, and the mixture was centrifuged for 3 min at 800×*g*. The pellet was mixed with 30 µL of 1% (w/v) bovine serum albumin in PBS, and the mixture was incubated on ice for 1 h for blocking. After blocking, the solution was for 3 min at 800×*g*, and the resultant pellet was mixed with Rabbit Anti-RbcL solution (1:1000 dilution in PBS; AS03 037A, Vännäs, Sweden). Then, the mixture was incubated at 277 K for 12 h. After centrifugation for 3 min at 800×*g*, the pellet was collected and resuspended in 100 µL of PBS. The cell pellets were collected after centrifugation for 3 min at 800×*g*, and resuspended in PBS with Anti-rabbit Alexa Fluor 594 (1:1000 dilution; Thermo Fisher Scientific, MA, USA). After a 1-h incubation for in the dark, cells were transferred on slides and stained with DAPI (0.1 μg/mL in PBS) Roche, Basel, Switzerland). The fluorescence images were captured using a FV1200 confocal laser microscope (Olympus, Tokyo, Japan) equipped with a GaAsP detector (Olympus, Tokyo, Japan).

### Specimen preparation for XFEL-XDI

For XFEL-XDI experiments, frozen-hydrated cells were prepared as described previously^35^ (Fig. 1). We used a custom-made specimen disk with nine windows (1 × 1 mm^2^ each), which was covered by a 100-nm thick silicon nitride (SiN) membrane (Norcada, Canada) (Fig. 1). To increase cell adhesion affinity, SiN membranes were coated with poly-l-lysine (Sigma Aldrich, USA) after deposition of a 40-nm thick carbon layer. The procedure of specimen preparation is briefly summarized below.

A 2-μL droplet of cell suspension was applied to a specimen disk in the interior of a chamber, the relative humidity in which was kept at higher than 90% to avoid drying^35,54^. After waiting for a few minutes to allow cell adhesion to SiN membrane, the excess suspension was removed using a filter paper or a MS-A100 spin coater (Mikasa, Japan). The average number density of cells on the membrane (approximately eight cells per 10 × 10 μm^2^) was monitored using an IX71 optical microscope (Olympus, Japan). Each specimen disk was flash-cooled in liquid ethane and stored in liquid nitrogen until their use in the experiments.

### XFEL-XDI experiment and data processing

We performed XFEL-XDI experiments using the TAKASAGO-6 diffraction apparatus^24^ in the experimental hutch 4 of BL3 or hutch 3 of BL2 in SACLA^55^ (Fig. 1). X-ray pulses with a wavelength of 0.225 nm were focused by a Kirkpatrick-Baez mirror optics to give an intensity of 10^10–11^ photons per 2 × 2 μm^2^ (full width at half-maximum) at each 10-fs pulse^56^. The diffraction apparatus was placed so that the specimen positioned within the focal spot of X-ray pulses. The transverse coherence of focused X-ray pulses was monitored to be higher than 0.99 using the method reported previously^57^.

The specimen disks were transferred from a liquid nitrogen bath to a cryogenic stage inside a vacuum chamber of the diffraction apparatus, and kept at 77 K. Each specimen disk was scanned at a speed of 25 μm/33 ms by a high-speed translation stage in synch with the arrival of X-ray pulses provided at a repetition rate of 30 Hz, because single X-ray pulses degrade specimens within approximately 10 μm from the center.

Diffraction patterns were recorded by two multi-port charge coupled device (MPCCD) detectors in a tandem arrangement^58^. A detector composed of eight CCD panels (MPCCD-octal) and another composed of two panels (MPCCD-Dual) were set at 1.6 m and 3.2 m downstream the specimen position, respectively. The MPCCD-Octal detector recorded diffraction patterns in a resolution range of 4.8 to 142.9 μm^−1^. Small angle diffraction passing through the aperture of the MPCCD-Octal detector was detected by the MPCCD-Dual detector. An aluminum attenuator with a thickness of 30 μm and a direct beamstop of 2 × 2 mm^2^ were placed in front of the MPCCD-Dual detector. As a result, diffraction patterns in a resolution range of 2.0 to 142.9 μm^−1^ could be collected with a dynamic range of approximately 10^5^ photons/detector pixel.

The diffraction patterns recorded by the two detectors were automatically merged by the G-SITTENNO program suite^59,60^ implemented on a SACLA HPC supercomputer. The patterns were merged into a single file using a pattern-matching algorithm to estimate the positions of direct beams. We selected diffraction patterns with high signal-to-noise ratios by assessing intensities, resolutions, and the centrosymmetry of diffraction patterns.

### Retrieval of projection electron density maps

All PR calculations were executed on a Mini-K super computer and a SACLA HPC super computer. Since PR calculations for a diffraction pattern do not always give correct electron density maps due to the lack of diffraction pattern hidden by the beam stop and Poisson noise in X-ray detection. We extracted probable PR maps from a number of independent PR calculations for each diffraction pattern through the procedures described below.

First, to estimate the shape of each cell, we performed 500 independent PR calculations for each diffraction pattern with a resolution of up to 20 μm^−1^, corresponding to 50 nm in real space, by using the hybrid-input–output (HIO) algorithm^22^ in combination with the shrink-wrap algorithm^61^. Then, we selected the most probable shape (support) resembling a circle with an approximate diameter of 1 μm after applying the multivariate analysis to the 500 maps^62^. Next, we performed 500 independent PR calculations to determine the electron density inside the selected support by using the oversampling smoothness (OSS) algorithm^63^ at a resolution of 40 μm^−1^ (corresponding to 25 nm in real space). The real-space constraint used is

2 $$ \begin{aligned} \rho_{k}^{\prime \prime} \left( {\mathbf{r}} \right) & = \left\{ {\begin{array}{ll} {\rho_{k}^{\prime} \left( {\mathbf{r}} \right)} & \quad {{\mathbf{r}} \in {\text{Support}}\;\;{\text{and}}\;\rho_{k}^{\prime} \left( {\mathbf{r}} \right) \ge 0,\;} \\ {\rho_{k} \left( {\mathbf{r}} \right) - \beta \rho_{k}^{\prime} \left( {\mathbf{r}} \right)} & \quad {{\text{otherwise}},} \\ \end{array} } \right. \hfill \\ \rho_{k + 1} \left( {\mathbf{r}} \right) & = \left\{ {\begin{array}{ll} {\rho_{k}^{\prime \prime} \left( {\mathbf{r}} \right)} & \quad {{\mathbf{r}} \in {\text{Support,}}} \\ {{\text{FT}}^{ - 1} \left[ {G_{k}^{\prime \prime} \left( {\mathbf{S}} \right)W\left( {\mathbf{S}} \right)} \right]} & \quad {{\mathbf{r}} \notin {\text{Support,}}} \\ \end{array} } \right. \hfill \\ W\left( {\mathbf{S}} \right) & = \exp \left[ { - \frac{1}{2}\left( {\frac{{\mathbf{S}}}{\alpha }} \right)^{2} } \right] \hfill \\ \end{aligned} $$

where $\rho_{k} \left( {\mathbf{r}} \right)$ is the map at the beginning of the *k*-th HIO-cycle, and $\rho_{k}^{\prime} \left( {\mathbf{r}} \right)$ is the inverse Fourier transform (FT^−1^) of the structure factor with the observed amplitude and phase calculated from $\rho_{k} \left( {\mathbf{r}} \right)$. $\rho_{k + 1} \left( {\mathbf{r}} \right)$ is an electron density map generated for the next cycle. *β* is the weight parameter fixed as 0.9 through HIO iterations. $G_{k}^{\prime\prime} \left( {\mathbf{S}} \right)$ is the Fourier transform of $\rho_{k}^{\prime\prime} \left( {\mathbf{r}} \right)$. For maps composed of *j* × *j* pixels, parameter *α* is linearly decreased from *j* to 1/*j* after every 1000 iterations. We carried out 20,000 OSS iterations by varying *α* for 20 times.

### Selection of diffraction patterns and projection electron density maps in phase-retrieval calculations

Here, we show how we select high quality data from a large number of datasets. We first selected diffraction patterns according to SN ratio at high-angle region. Diffraction patterns selected automatically by our software suite^59,60^, in which at least two detector pixels received more than four photons at spatial frequency 30 μm^−1^. And then, we selected manually diffraction patterns with clear speckle peaks in size of 1.3 μm^−1^ up to spatial frequency of 40 μm^−1^.

Next, we selected electron density maps after PR calculations. To find the most probable set of retrieved density maps, we used a score defined as follows:

3 $$ T_{{{\text{ij}}}} = \frac{{\sum\limits_{x,y} {\left| {\rho_{{\text{i}}} \left( {x,y} \right) - \rho_{j} \left( {x,y} \right)} \right|} }}{{\sum\limits_{x,y} {\left| {\rho_{{\text{i}}} \left( {x,y} \right) + \rho_{j} \left( {x,y} \right)} \right|} }}, $$

where $\rho_{{\text{i}}} \left( {x,y} \right)$ is the electron density at a pixel position $\left( {x,y} \right)$ of the *i*-th map. We previously reported that this score provides an accurate criterion for the selection of the probable PR maps from a large number of independently retrieved maps^64^. From the frequency distribution of peak position of $T_{{{\text{ij}}}}$ (SI appendix, Figure S2C), we adopted PR maps displaying the frequency distribution of the *T*_ij_ scores calculated for a set of 500 maps displayed a single peak centered at less than 0.2, which can effectively select the most probable PR maps in solution space as described in SI appendix Fig. S3. Then, the most probable map was obtained by averaging 10 PR maps from the lowest $T_{{{\text{ij}}}}$ values. According to this criterion, we reject PR maps which may be reconstructed under poor convergence on PR calculation (SI appendix, Fig. S2G).

Crystallographic *R*-factor of the most probable map was also calculated as follows:

4 $$ R_{{\text{F}}} = \frac{{\sum\limits_{{\varvec{S}}} {\left| {\left| {F_{{{\text{obs}}}} \left( {\varvec{S}} \right)} \right| - K\left| {F_{{{\text{calc}}}} \left( {\varvec{S}} \right)} \right|} \right|} }}{{\sum\limits_{{\varvec{S}}} {\left| {F_{{{\text{obs}}}} \left( {\varvec{S}} \right)} \right|} }}, $$

where $\left| {F_{{{\text{obs}}}} \left( {\varvec{S}} \right)} \right|$ and $\left| {F_{{{\text{calc}}}} \left( {\varvec{S}} \right)} \right|$ refer to the structure amplitude observed experimentally and that calculated from the most probable map at a scattering vector ${\varvec{S}}$, respectively. *K* is a scale factor.

We also used phase retrieval transfer function (PRTF) to evaluate resolution of PR maps^62^, as following equation,

5 $$ \begin{aligned} PRTF (\mathbf{S}) = \frac{{\left| {\left\langle {F_{{{\text{calc}}}} \left( {\mathbf{S}} \right)} \right\rangle } \right|}}{{\left| {F_{{{\text{obs}}}} \left( {\mathbf{S}} \right)} \right|}}, \hfill \\ \left\langle {F_{{{\text{calc}}}} \left( {\mathbf{S}} \right)} \right\rangle  = {\text{FT}}\left[ {\frac{1}{M}\sum\limits_{m = 1}^{M} {\rho_{m} \left( {\mathbf{r}} \right)} } \right] \hfill \\ \end{aligned} $$

where FT represents the Fourier transform, and $\left| {\left\langle {F_{{{\text{calc}}}} \left( {\mathbf{S}} \right)} \right\rangle } \right|$ is the structure amplitude of a map obtained by averaging *M* maps with the electron density $\rho_{m} \left( {\mathbf{r}} \right)$. We defined the effective resolution of the PR maps, where the radially averaged ${\text{PRTF}} \left( {\text{S}} \right)$ drops to 0.5^62,65^. We used both the *R*-factor and effective resolution by PRTF as a post filter to select high quality PR maps, but we found no maps displaying substantially worse values of them (SI appendix, Fig. S2D,E). Thus, we adopted all the 293 reconstructions with $T_{{{\text{ij}}}}$ < 0.2 for the following 3D reconstruction.

### Reconstruction of 3D map

XFEL pulses fluctuated in the intensities. In addition, in our irradiation method (Fig. 1), as the geometrical overlap of XFEL pulses and cells varied shot-by-shot, it is very difficult to experimentally evaluate electron densities of each PR map as the absolute scale. Therefore, prior to 3D reconstruction, projection maps were normalized with respect to the sums of the electron densities in each. 3D reconstruction from projection maps was carried out using EMAN program suite implementing the back-projection method^66^ according to the procedure reported in our simulation studies previously^67,68^.

At the first step, a Gaussian low-pass filter was applied to projection maps by using the *proc2d* protocol to reduce influences from the fine structures. An initial 3D density map was constructed using the *startAny* protocol without a symmetry restraint. Then, the density map was improved using the *refine* protocol through a stepwise variation of a Gaussian low-pass filter. We assumed 332 orientations at an angular step of 8.0º to survey angular space evenly. Each projection map was assigned to one orientation. Finally, a 3D map was obtained by applying the *refine* protocol to the projection maps without low-pass filtering.

The effective resolution of a reconstructed 3D map was evaluated by the Fourier shell correlation (FSC) for a given resolution shell *S′* as

6 $$ FSC\left( {S^{\prime}} \right) = \frac{{{\text{Re}} \sum\nolimits_{{S \in S^{\prime}}} {F_{1} \left( S \right)F_{2}^{*} \left( S \right)} }}{{\sqrt {\sum\nolimits_{{S \in S^{\prime}}} {\left| {F_{1} \left( S \right)} \right|}^{2} \sum\nolimits_{{S \in S^{\prime}}} {\left| {F_{2} \left( S \right)} \right|}^{2} } }}, $$

where *F*_1_(*S*) (or *F*_2_(*S*)) is the structure factor of the 3D map from the half set of the projection maps at a resolution shell of *S*. An effective resolution was defined as that the FSC value dropped to 0.143^69^.

## Supplementary Information

Supplementary Information.
