# Supplementary material for: Common architectures in cyanobacteria Prochlorococcus cells visualized by X-ray diffraction imaging using X-ray free electron laser
Source: Sci Rep. 2021 Feb 16;11:3877. doi: 10.1038/s41598-021-83401-y (PMC7886902; doi:10.1038/s41598-021-83401-y)
Supplement: Supplementary file 1 — Supplementary Information. [file 41598_2021_83401_MOESM1_ESM.pdf]

Supplementary Information

Common architectures in cyanobacteria *Prochlorococcus* cells visualized by Xray diffraction imaging using Xray free electron laser

Amane Kobayashi, Yuki Takayama, Takeshi Hirakawa, Koji Okajima, Mao Oide, Tomotaka Oroguchi, Yayoi Inui, Masaki Yamamoto, Sachihiro Matsunaga, and Masayoshi Nakasako,*

* To whom correspondence should be addressed. Phone: +81-45-566-1713. Fax: +81-45-566-1672.

E-mail: nakasako@phys.keio.ac.jp.

S1. Flow cytometry of Cyanobacteria cells before specimen preparation for XFEL-XDI experiments

To examine amounts of cells in the interphase and mitotic phase in a culture, we performed flow cytometry as described previously (Watanabe *et al.*, 2012). First, culture medium was mixed with Tween 20 (Wako, Japan) and glutaraldehyde (Wako, Japan) at a final concentration of 0.1% (v/v) and 1% (w/v), respectively. The mixture was incubated for 30 min at 277 K, and was washed with phosphate buffered saline (PBS) three times by centrifugation at 25,000 × *g* for 5 min. Then, the cell pellet was frozen in liquid nitrogen. After thawing, DNA in the cells was stained with 10 μM SYTOX Green (Invitrogen, Carlsbad, CA, USA) by incubating for 12 h at 277 K in the dark. The medium was diluted with PBS buffer so that the final concentration of SYTOX Green was 0.2 μM. Flow cytometry for the prepared specimen was carried out by using a CytoFLEX instrument (Beckman Coulter, USA).

Figure S1A shows histograms of the amount of DNA included in *Prochlorococcus* cells in four different XFEL-XDI experiments. Each histogram comprises a prominent peak (red) and two small maxima (green and blue). The position of the prominent peak indicates that the cells were in the interphase. The cells appearing in the small second maxima in blue were in the mitotic phase. The particles in the third group were contamination with sizes of approximately 1 × 5 μm^2 (Fig. S1B).

In addition, even when the cells and contamination in the small maxima in flow cytometry were contained in a flash-cooled XFEL-XDI specimen, their diffraction patterns were excluded from structure analyses, because the sizes and shapes of speckle peaks were significantly smaller than those for *Prochlorococcus* cells in the interphase. In addition, mitotic cells, which contain almost doubled cell contents from those of

interphase cells, give diffraction patterns with strong intensities exceeding the overflow limit of detector pixels.

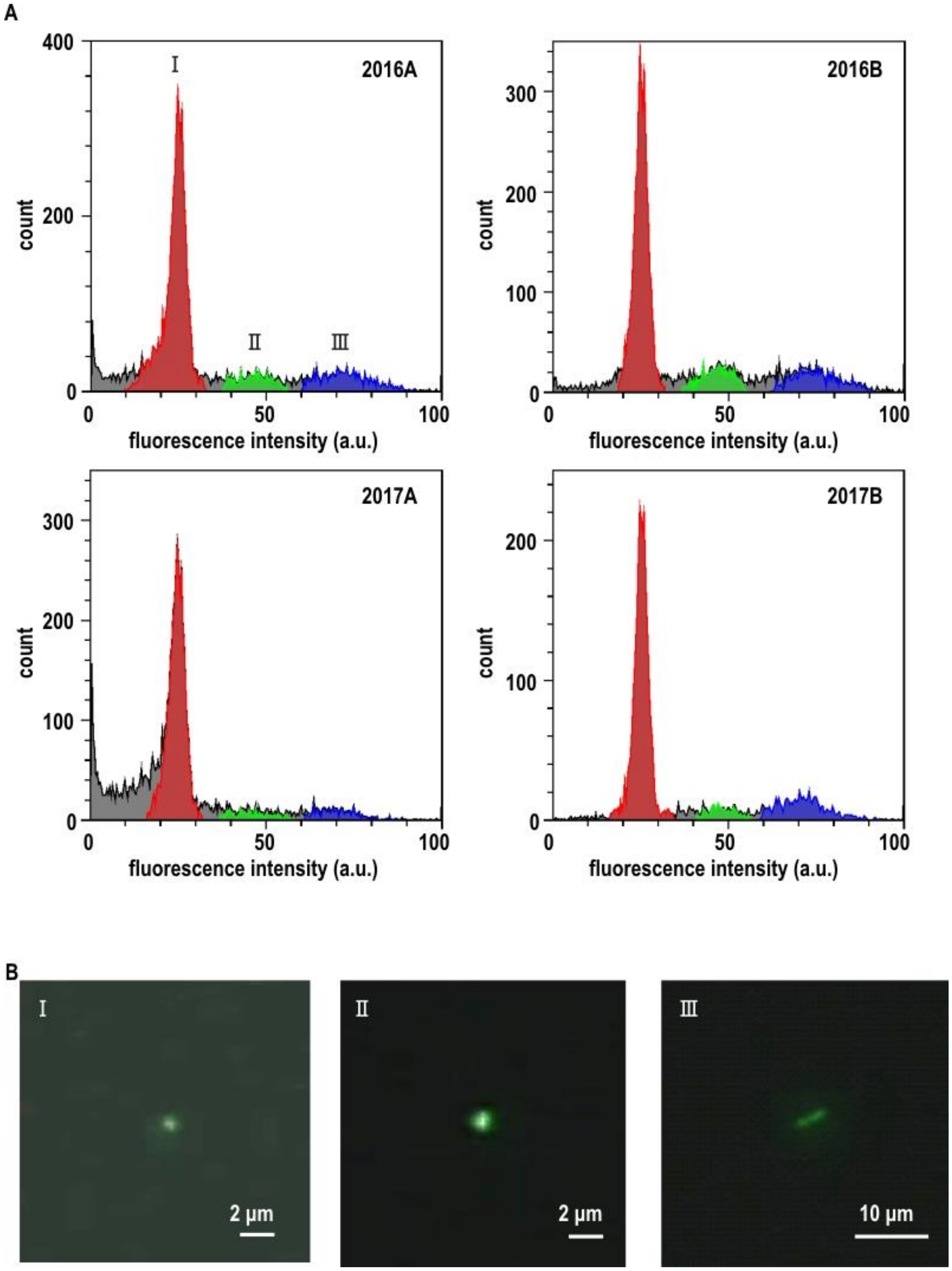

Figure S1

(A) Histograms of DNA content in *Prochlorococcus marinus* MED4 cells in four different XFEL-XDI experiments (2016A, 2016B, 2017A, and 2017B). The ratio of cells in interphase (red in the histograms), mitotic phase (green), and cells of other species (blue) was 100:6:15 in 2016A specimen, 100:8:23 in 2016B, 100:4:10 in 2017A, and 100:4:26 in 2017B. (B) Fluorescence images of cells isolated from regions I, II, and III using a cell sorter (MolFlo™ XDP IntelliSort II, Beckman Coulter, USA) in the 2016A specimen of panel A. The images were taken using IX71 (Olympas, Japan).

References

S. Watanabe, R. Ohbayashi, Y. Shiwa, A. Noda, Y. Kanesaki, T. Chibazakura & H. Yoshikawa. Light-dependent and asynchronous replication of cyanobacterial multi-copy chromosomes. *Mol. Microbiol.* **83**, 856–865 (2012).

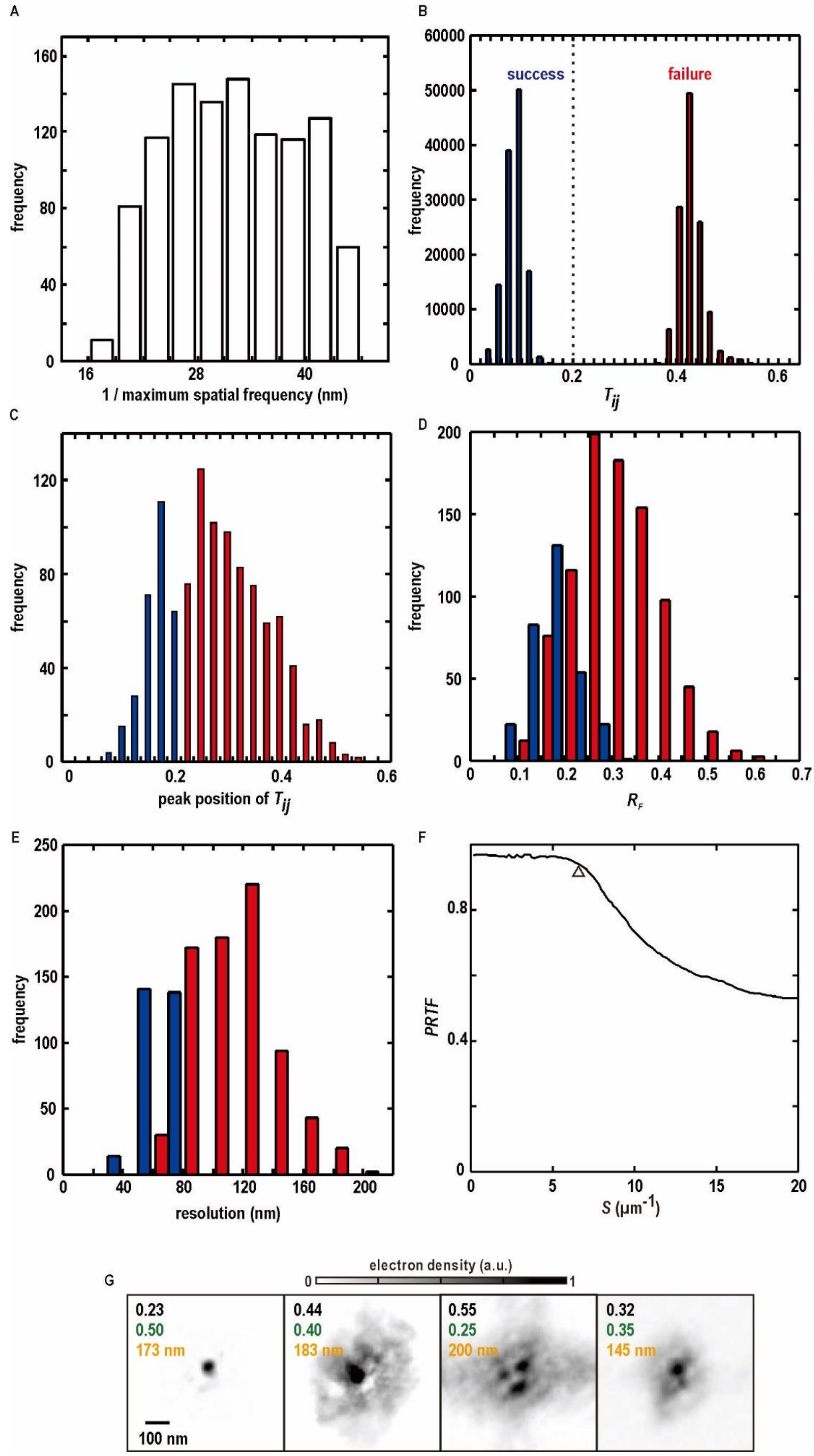
A
frequency
1 / maximum spatial frequency (nm)
B
success
failure
frequency
T_{ij}
C
frequency
peak position of T_{ij}
D
frequency
R_F
E
frequency
resolution (nm)
F
PRTF
S (μm^{-1})
G
electron density (a.u.)
0.23
0.50
173 nm
100 nm
0.44
0.40
183 nm
0.55
0.25
200 nm
0.32
0.35
145 nm

Figure S2

(A) Frequency distribution of maximum spatial frequency in real space among 1,061 diffraction patterns, which is defined as the highest-frequency shell including at least three detector pixels with more than four photons (Sekiguchi *et al.*, 2014). (B) Representative examples of frequency distribution of T_{ij} values (Sekiguchi *et al.*, 2017) in two sets of 500 phase-retrieval (PR) maps. In this and subsequent panels, the number of maps judged as success and failure are displayed by blue and red bars. (C) Frequency distribution regarding the peak positions in the frequency distributions of T_{ij} values, as panel (B), in the PR calculations for 1,061 diffraction patterns. The distribution displayed a valley at T_{ij} ~ 0.2. Therefore, we judge a set of PR calculations as success, when the distribution peak is located at T_{ij} smaller than 0.2, and otherwise as failure. Frequency distributions of crystallographic *R*-factors (D) and the resolution estimated by the phase-retrieval transfer function (PRTF) (E) of PR maps. (F) An averaged PRTF curve among 293 PR maps. The arrowhead indicates the effective resolution evaluated by FSC. The FSC value is kept at larger than 0.9 up to a resolution of 136 nm. (G) Representative failure PR maps. Inset numbers at upper left indicates peak position of T_{ij} (black), *R*-factors (green), and efficient resolution evaluated by PRTF (orange).

References

Y. Sekiguchi, M. Yamamoto, T. Oroguchi, Y. Takayama, S. Suzuki, & M. Nakasako, IDATEN and G-SITENNO: GUI-assisted software for coherent X-ray diffraction imaging experiments and data analyses at SACLA. *J. Synchrotron Radiat.* **21**, 1378–83 (2014).

Y. Sekiguchi, S. Hashimoto, A. Kobayashi, T. Oroguchi & M. Nakasako, A protocol for searching the most probable phase-retrieved maps in coherent X-ray diffraction imaging by exploiting the relationship between convergence of the retrieved phase and success of calculation. *J. Synchrotron Radiat.* **24**, 1024–1038 (2017).

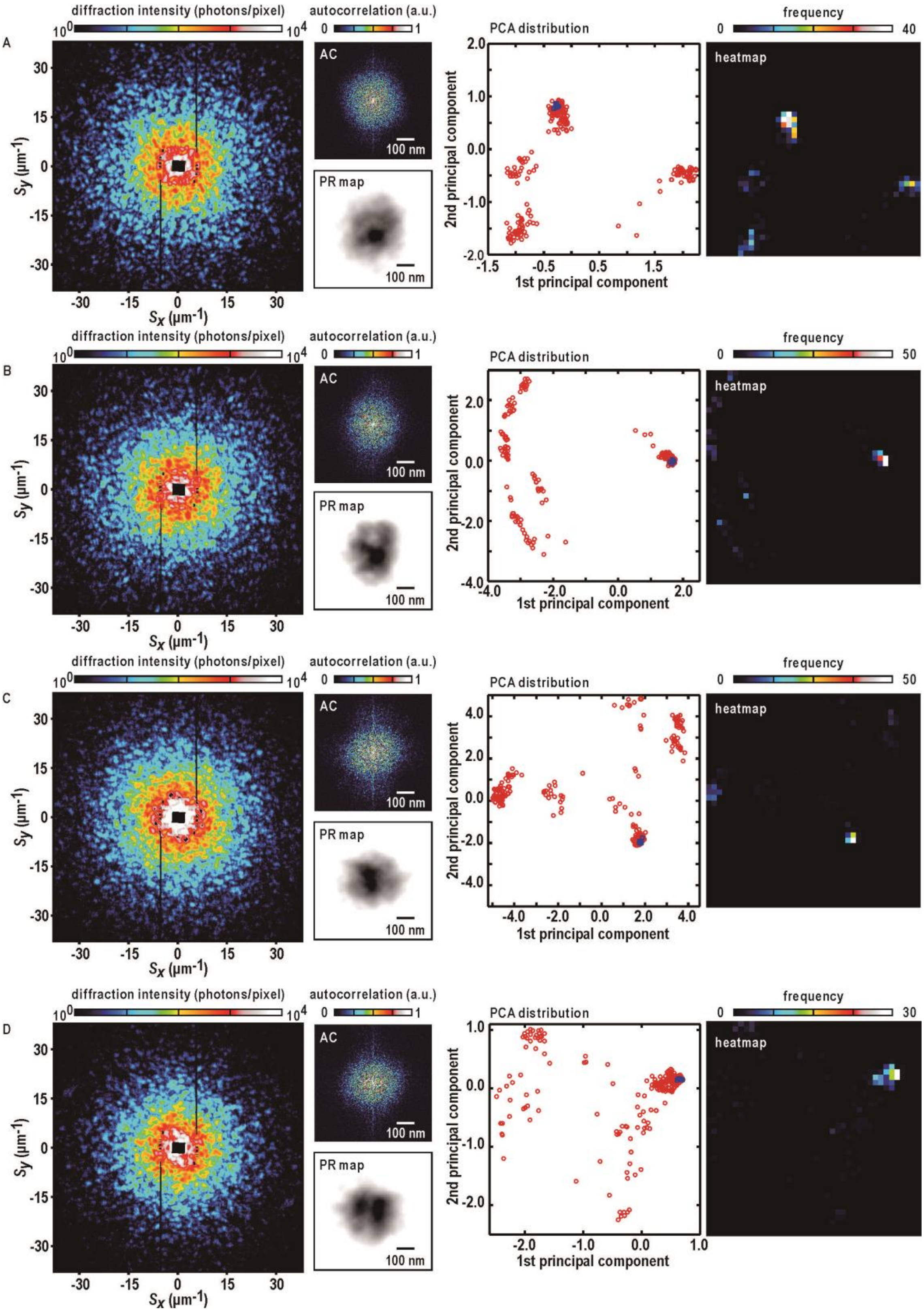

Figure S3

The left column shows representative examples of diffraction patterns, their

autocorrelation function used for estimate the dimension and shape of particle (labeled AC in the middle column), and PR maps. To mitigate distortion in the autocorrelation due to missing low-resolution data, we applied a filter introduced in Chapman et al 2006. The plots in the right column displays the distribution of 10 PR maps with the lowest T_{ij} values (blue dots) and other 490 maps (red) on the plane spanned by first and second principal conponents for 500 PR maps (Sekiguchi et al. 2016). The frequency distributions in the plot is presented as heat maps. It is clear that the positions of 10 PR maps in the plot almost overlap with the most frequent region in the heatmaps.

References

H. N. Chapman, A. Barty, S. Marchesini, A. Noy. & S. P. Hau-Riege, High-resolution *ab* initio three-dimensional x-ray diffraction microscopy, *J. Opt. Soc. Am. A,* **23**, 1179 (2006).

Y. Sekiguchi, T. Oroguchi, & M. Nakasako, Classification and assessment of retrieved electron density maps in coherent X-ray diffraction imaging using multivariate analysis. *J. Synch. Rad.* **23**, 312–323 (2016).

S2. Electron density contrast in projection maps

In our XDI experiments, cells are embedded in vitreous ice. At resolution of several tens nanometers, projected electron density distribution of vitreous ice can be approximated to be constant (ρ_{sol}). Then, projected electron distribution $\rho(\vec{r})$ (Fig. S4) is expressed by ρ_{sol} and electron density $\rho_{\mathrm{ex}}(\vec{r})$ in a cell excess from ρ_{sol} as the following equation,

$$\rho(\vec{r}) = \rho_{\mathrm{sol}} + \rho_{\mathrm{ex}}(\vec{r})$$

$$\rho_{\mathrm{ex}}(\vec{r}) = \Delta\rho\,\rho_{\mathrm{C}}(\vec{r}) + \rho_{\mathrm{F}}(\vec{r}), \quad \Delta\rho = \bar{\rho} - \rho_{\mathrm{sol}}$$

where $\rho_{\mathrm{C}}(\vec{r})$ expresses the shape of the cell with the average electron density $\bar{\rho}$, and takes 1 inside and 0 outside the cell. $\rho_{\mathrm{F}}(\vec{r})$ describes the spatial fluctuation of electron density from the average $\bar{\rho}$. The diffraction intensity at a scattering vector $\vec{S}$, $I(\vec{S})$ is expressed as

$$I\left(\vec{S}\right) \propto 4\pi^2 \rho_{\mathrm{sol}}{}^2 \delta\left(\vec{S}\right)^2 + \Delta\rho^2 \left| F_{\mathrm{C}}\left(\vec{S}\right) \right|^2 + \Delta\rho\, F_{\mathrm{C}}^*\left(\vec{S}\right) F_{\mathrm{F}}\left(\vec{S}\right) + \Delta\rho\, F_{\mathrm{F}}^*\left(\vec{r}\right) F_{\mathrm{C}}\left(\vec{S}\right) + \left| F_{\mathrm{F}}\left(\vec{S}\right) \right|^2,$$

where $\delta(\vec{S})$ is Dirac delta function. $F_{\mathrm{C}}(\vec{S})$ and $F_{\mathrm{F}}(\vec{S})$ are the structure factors of $\rho_{\mathrm{C}}(\vec{r})$ and $\rho_{\mathrm{F}}(\vec{r})$, respectively. Because the first term is zero except for $\vec{S} = 0$, X-rays diffracted by solvent with ρ_{sol} outside the cell within the beam size of incident X-rays are hidden by a beam stop. Therefore, electron density outside the cell is zero in the projection maps reconstructed from diffraction patterns collected in experiments, that is

why shapes of projection maps with finite size can be defined in phase retrieval procedure. In addition, the diffraction intensity is dominated by $\left|F_F\left(\vec{S}\right)\right|^2$ when $\Delta\rho$ becomes small. For instance, average electron density value of nucleic acids, proteins, and water are 0.55, 0.42, and 0.33 electrons Å^{-3}, respectively [Stuhrmann & Miller, 1978]. Therefore, the projection maps of cells in solvent or vitreous ice display internal structures with high electron density region rather than overall shapes in the projection maps.

The influence of electron density contrast was examined in our previous simulation studies regarding X-ray diffraction imaging of macromolecules buried in vitreous ice or water (Kodama & Nakasako, 2011; Oroguchi & Nakasako, 2013). As demonstrated in the studies, the border of macromolecules is blurred by electron density contrast between macromolecules and vitreous ice. The same effect would occur in the XFEL study on cells.

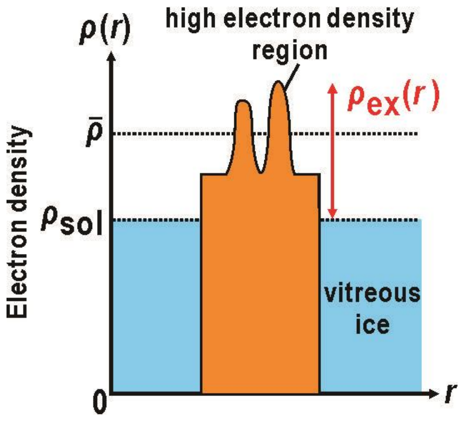

Figure S4

Schematic illustration of electron density distribution in case of a cell in solvent or

vitreous ice.

Reference

H. B. Stuhrmann, & A. Miller, Small-angle scattering of biological structures. *J Appl Crystallogr* **11**, 325–345 (1978).

W. Kodama, M. Nakasako,. Application of real-space three-dimensional image reconstruction method in the structural analysis of non-crystalline biological particles in water envelop by coherent X-ray diffraction microscopy. *Phys. Rev. E* **84**, 021902 (15 pages) (2011).

T. Oroguchi, & M. Nakasako, Three-dimensional structure determination protocol for noncrystalline biomolecules using x-ray free-electron laser diffraction imaging. *Phys. Rev. E* **87**, 022712 (15 pages) (2013).

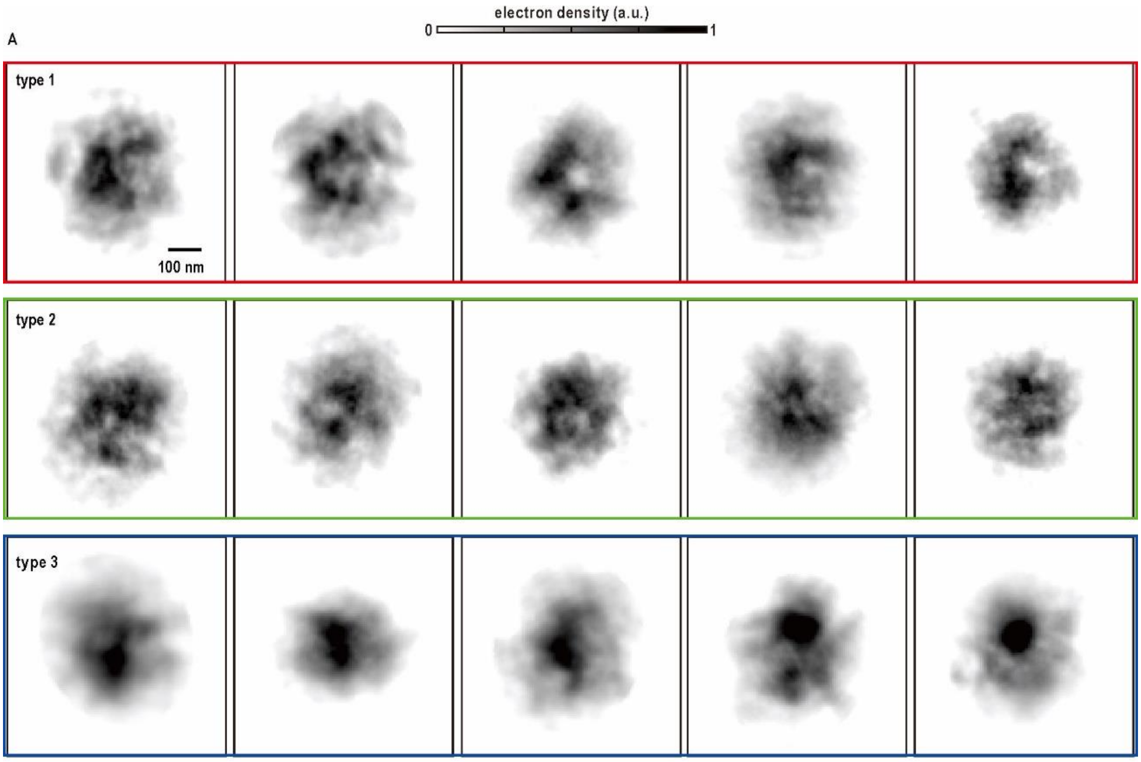

Figure S5

Representative projection maps classified into types 1-3.

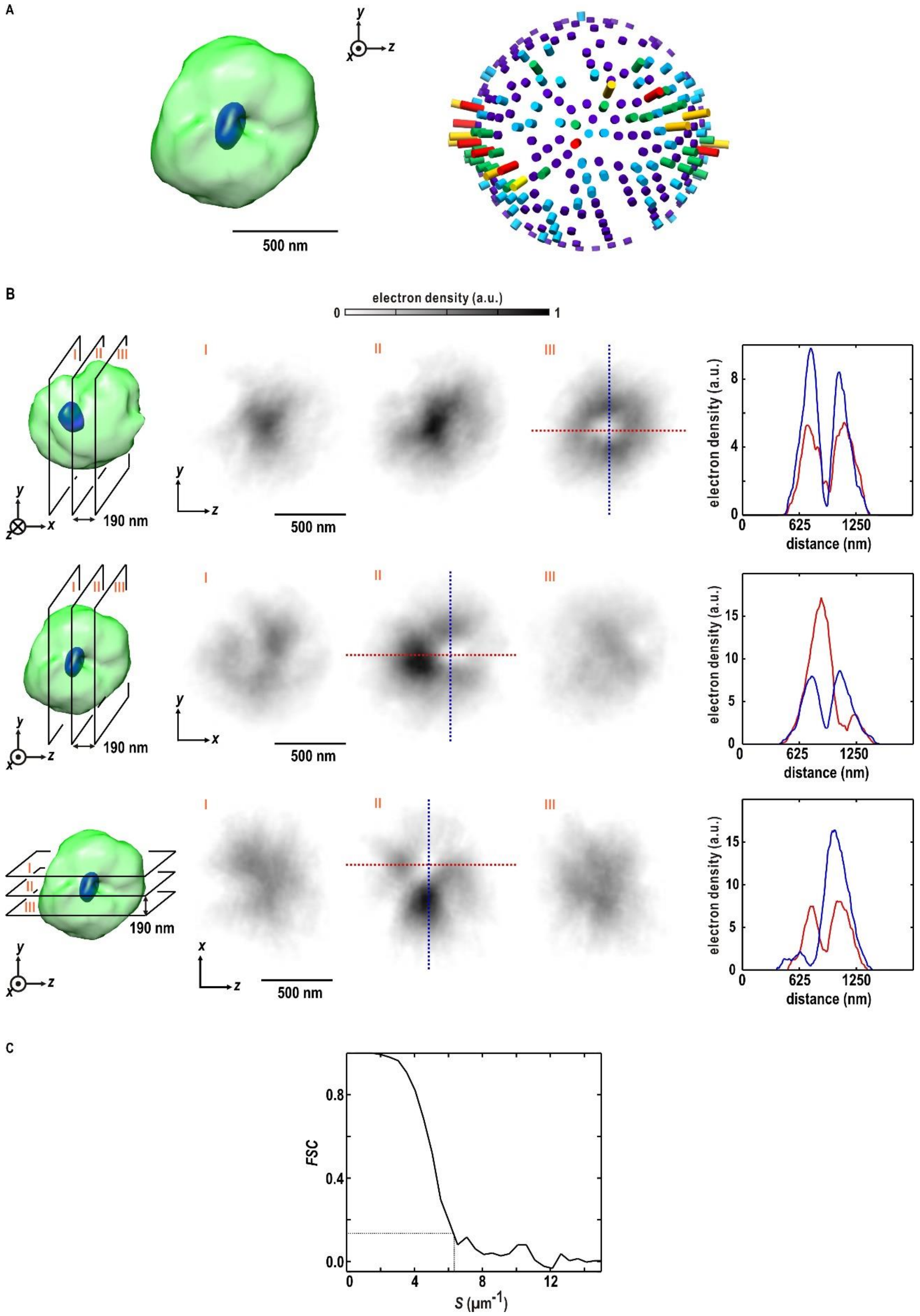
A
y
x
z
500 nm
B
electron density (a.u.)
0
1
I II III
190 nm
500 nm
electron density (a.u.)
distance (nm)
0
625
1250
C
FSC
S (μm-1)
0.8
0.4
0.0
0
4
8
12

Figure S6

(A) Estimated angular distribution of 293 projection maps (right panel) at the view of a reconstructed 3D map (left). Height of each cylinder indicates the number of projection maps used for the 3D reconstruction at the orientation. The number of maps is also indicated by the color of each cylinders (purple, cyan, green, red and orange cylinders mean the use of one to five maps in each orientation, respectively). (B) A set of slices of the 3D map viewed along the three principal directions. In the right column, the electron-density profiles crossing the void region are plotted along and normal to the preferred orientation directions. (C) A plot of Fourier shell correlation (FSC) values of the reconstructed 3D map. The effective resolution is determined by a threshold of 0.143 [Rosenthal and Henderson, 2003].

S3. Comparison of reconstructed projection maps and observed structure amplitude with those from the 3D map

Here we compare the phase-retrieved (PR) maps with the projection maps calculated from the reconstructed 3D map (Figs. S7A and B). Each projection map was calculated by projecting the 3D map using the orientation parameters of the PR maps, which was determined in the single particle analysis. To evaluate the similarity between the PR and projection maps, we used zero-mean normalized cross-correlation (ZNCC) function defined as

$$\mathrm{ZNCC}=\frac{\sum_{y}\sum_{x}\left(\rho(x,y)-\overline{\rho}\right)\left(\rho_{\mathrm{proj\_3D}}(x,y)-\overline{\rho_{\mathrm{calc}}}\right)}{\sqrt{\sum_{y}\sum_{x}\left(\rho(x,y)-\overline{\rho}\right)^{2}\sum_{y}\sum_{x}\left(\rho_{\mathrm{proj\_3D}}(x,y)-\overline{\rho_{\mathrm{proj\_3D}}}\right)^{2}}},$$

where $\rho(x,y)$ and $\rho_{\mathrm{proj\_3D}}(x,y)$ are electron density of a projection map and calculated one at pixel position (x,y).

We also compared the observed structure amplitude with that of the projection map calculated from the 3D map, which we here treated as ensemble average of PR maps, by using the phase retrieval transfer function (PRTF_3D) as

$$\mathrm{PRTF\_3D}(\mathbf{S})=\frac{\left|F_{\mathrm{proj\_3D}}(\mathbf{S})\right|}{\left|F_{\mathrm{obs}}(\mathbf{S})\right|},$$

$$F_{\mathrm{proj\_3D}}(\mathbf{S})=\mathrm{FT}\left[\rho_{\mathrm{proj\_3D}}(x,y)\right],$$

where $\left|F_{\mathrm{obs}}(\mathbf{S})\right|$ is the experimentally observed structure amplitude, and $\left|F_{\mathrm{proj\_3D}}(\mathbf{S})\right|$ is that calculated by Fourier transformation (FT) of the projection map $\rho_{\mathrm{proj\_3D}}(x,y)$ from the 3D map at a scattering vector $\boldsymbol{S}$. Figure S7C shows the PRTF_3D curve averaged over the ensemble of the projection maps. The curve kept high values of larger

than 0.8 beyond the 3D resolution estimated by FSC analysis (Fig. S6B). The high PRTF value indicates that the 3D map is almost free from the phase retrieval error up to the resolution.

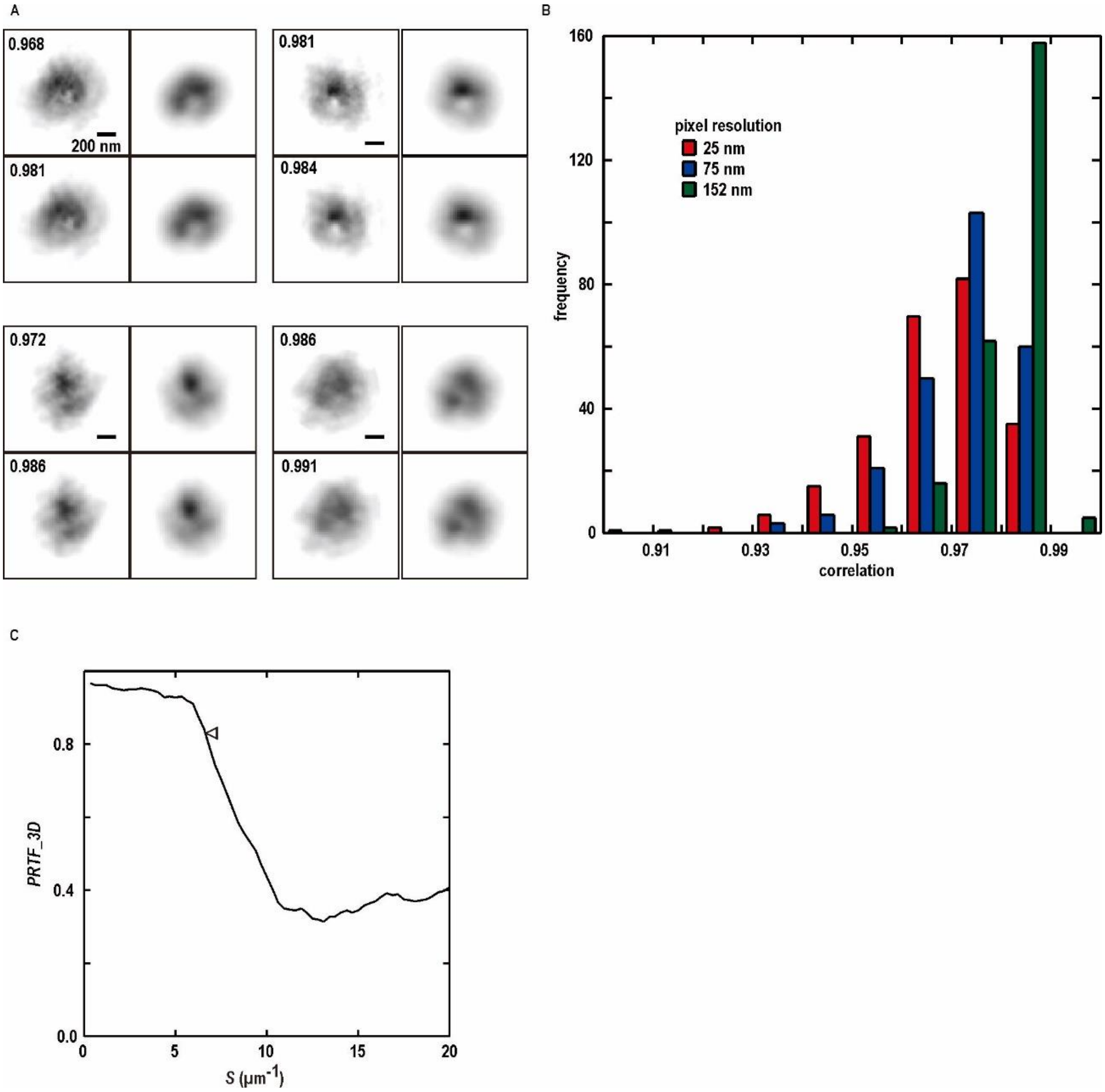

Figure S7

(A) Examples of comparison for four representative sets of the PR maps and projection maps calculated from the 3D map. Upper row in each panel shows a PR map (upper left) and the projection map along the direction of the determined orientation (upper right).

The pixel resolution is 25 nm. Maps in the lower row are projection maps, which are blurred from the maps in the upper row after adjusting the pixel resolution to 152 nm. Values labeled in the left maps are the correlation coefficient values between the PR and projection maps. (B) Frequency distribution of the correlation coefficients among 293 projection maps at pixel resolution at 25 nm (red bars), 75 nm (blue) and 152 nm (green). (C) PRTF_3D curve averaged among 293 sets of structure amplitudes. The arrowhead indicates the effective resolution evaluated by FSC.

Reference

P. Rosenthal & R. Henderson, Optimal Determination of Particle Orientation, Absolute Hand, and Contrast Loss in Single-particle Electron Cryomicroscopy. *J. Mol. Biol.* **333**, 721–745 (2003).

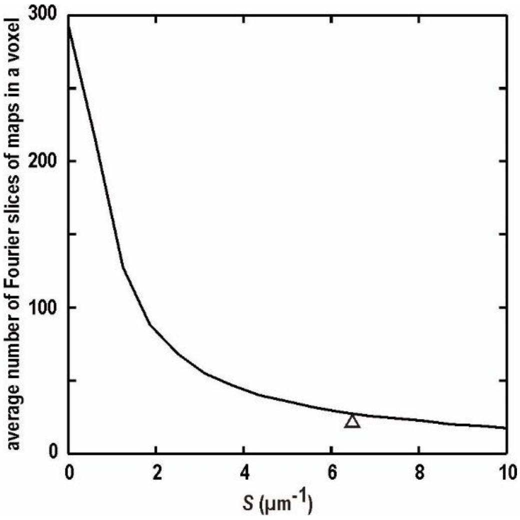

Figure S8

Averaged number of Fourier slices calculated by electron density maps in a voxel. The Fourier slices in 3D Fourier space were calculated by Fourier transformation of the projection maps from XFEL-XDI according to the Fourier slice theorem with the orientation determined by 3D reconstruction. The Fourier voxel size was 0.625 μm^{-1} corresponding to the Nyquist interval assuming the size of the cyanobacterial cells of 800 nm. The arrowhead indicates the effective resolution evaluated by FSC.
